# Brain temperature and free water increases after mild COVID-19 infection

**DOI:** 10.1038/s41598-024-57561-6

**Published:** 2024-03-28

**Authors:** Ayushe A. Sharma, Rodolphe Nenert, Adam M. Goodman, Jerzy P. Szaflarski

**Affiliations:** 1 Department of Neurology, UAB Epilepsy Center, University of Alabama at Birmingham (UAB), 1719 6th Avenue South, CIRC 312, Birmingham, AL 35294-0021 USA; 2 Department of Neurobiology, University of Alabama at Birmingham (UAB), Birmingham, AL USA; 3 Department of Neurosurgery, University of Alabama at Birmingham (UAB), Birmingham, AL USA; 4 University of Alabama at Birmingham Epilepsy Center (UABEC), Birmingham, AL USA

**Keywords:** COVID-19, Neuroinflammation, Magnetic resonance spectroscopic imaging, Brain thermometry, Post-acute sequelae of COVID-19, Neurology, Image processing

## Abstract

The pathophysiology underlying the post-acute sequelae of COVID-19 remains understudied and poorly understood, particularly in healthy adults with a history of mild infection. Chronic neuroinflammation may underlie these enduring symptoms, but studying neuroinflammatory phenomena in vivo is challenging, especially without a comparable pre-COVID-19 dataset. In this study, we present a unique dataset of 10 otherwise healthy individuals scanned before and after experiencing mild COVID-19. Two emerging MR-based methods were used to map pre- to post-COVID-19 brain temperature and free water changes. Post-COVID-19 brain temperature and free water increases, which are indirect biomarkers of neuroinflammation, were found in structures functionally associated with olfactory, cognitive, and memory processing. The largest pre- to post-COVID brain temperature increase was observed in the left olfactory tubercle (*p* = 0.007, 95% CI [0.48, 3.01]), with a mean increase of 1.75 °C. Notably, the olfactory tubercle is also the region of the primary olfactory cortex where participants with chronic olfactory dysfunction showed the most pronounced increases as compared to those without lingering olfactory dysfunction (adjusted *p*_FDR_ = 0.0189, 95% CI [1.42, 5.27]). These preliminary insights suggest a potential link between neuroinflammation and chronic cognitive and olfactory dysfunction following mild COVID-19, although further investigations are needed to improve our understanding of what underlies these phenomena.

## Introduction

Severe acute respiratory syndrome coronavirus 2 (SARS-CoV-2), the coronavirus strain responsible for coronavirus disease 2019 (COVID-19), has significantly impacted global health, with over 500 million documented cases worldwide. The serious, lasting neurological consequences of severe COVID-19 infections are well-documented in the neuroimaging literature, but even individuals with asymptomatic or mild COVID-19 can experience long-term effects on brain health^1–4^. These individuals can experience a spectrum of neurological symptoms, collectively referred to as “brain fog” by lay populations, including fatigue, mood changes, impaired cognition, and chronic loss of smell^4,5^. Fortunately, these symptoms typically resolve within three months^2^. Still, many individuals continue to experience these symptoms beyond the three-month mark, at which point they are said to suffer from the post-acute sequelae of COVID-19 (PASC) or “long COVID.” Subtle decreases in attention and memory are evident upon objective testing even in those who do not self-report PASC^6^. The mechanisms underlying these chronic symptoms are not fully understood, highlighting the need for more neuroimaging studies that investigate the neural substrates of PASC following mild COVID-19 infection.

Neuroimaging studies of COVID-19 have primarily focused on visualizing macroscopic abnormalities in hospitalized patients^7^. However, neuroimaging studies are increasingly investigating the more subtle, long-lasting changes thought to underlie chronic post-COVID symptoms in patients with mild infections^1–4^. Structural and diffusion imaging have been the primary techniques utilized in studies to date, with positron emission tomography findings reported in select case series. For example, a case–control positron emission tomography (PET) study found that three MRI-normal patients with PASC had impaired memory and executive function associated with reduced metabolism in the anterior and posterior cingulate cortex^8^. Additionally, patients with PASC have demonstrated lower connectivity between the orbitofrontal and cerebellar regions, accompanied by grey matter volume (GMV) reductions and cognitive dysfunction^9^. Retrospective and prospective studies have provided valuable information, but less is known about the neurological consequences of mild COVID-19 infections and individual pre- to post-COVID-19 changes. In a large-scale study conducted by the UK Biobank, 401 individuals were imaged before and after COVID-19 infection^10^. Results showed post-COVID-19 thinning of the orbitofrontal cortex and parahippocampal gyrus, microstructural damage in areas connected with the primary olfactory cortex, and overall brain volume reductions^10^. However, the study cohort was comprised of individuals aged between 51 and 81 years, making it difficult to distinguish between the cognitive effects of aging and of the virus itself^10^. Additionally, the study did not specifically focus on individuals with PASC following mild infection^10^.

Research on PASC in young, healthy individuals is limited. While chronic, low-level neuroinflammation is believed to be one of the mechanisms underlying PASC, few, if any, studies have investigated pre- vs. post-COVID-19 inflammatory phenomena in these patients. Emerging noninvasive techniques, such as magnetic resonance spectroscopic imaging and thermometry (MRSI-t) and neurite orientation dispersion and density imaging (NODDI), may allow indirectly mapping the consequences of neuroinflammation and may thus be useful for imaging the pathophysiology associated with chronic COVID-19 symptoms^4,11,12^. However, few studies have employed NODDI or similar multicompartment modeling techniques to detect post-COVID-19 increases in extracellular free water, an indicator of edema^13,14^. Although MRSI-t has potential value, it has not been used in any study of mild COVID-19 or its associated neurological manifestations. MRSI-t uniquely enables the calculation and mapping of brain temperature in every voxel of the brain, with brain temperature elevations potentially serving as a biomarker of neuroinflammatory activity^15,16^. Brain temperature is typically 0.5–1 °C higher than core body temperature^17,18^. However, when neuroinflammation impairs the brain's cooling mechanisms in a specific region or set of regions, temperatures in the corresponding tissue may increase by an additional 0.5 to 1 °C^19–24^. Moreover, localized increases in brain temperature are linked to increased leukocyte extravasation, cerebral edema, and disruption of the blood–brain barrier. Still, brain temperature mapping is in its early stages, and the exact threshold that distinguishes "abnormal" from "normal" brain temperature is still being defined. This is particularly challenging due to the numerous factors (age, sex, menstrual cycle, brain region, time of day) that may influence these values^25^. Given the subtle differences between "normal" and "abnormal" brain temperature, establishing a baseline is crucial. In our initial MRSI-t study, we observed consistent brain temperature values for each region of interest (ROI) when imaging healthy participants twice, with a 12-week interval between study visits and without tight control of variables (e.g., time of day) thought to impact brain temperature measurements^16^. The coefficient of variation for repeated measures (COVrep) for the 47 ROIs ranged from 0.81% to 3.08%, with a mean COVrep of 1.92%^16^. Most ROIs had a COVrep below 2.0%, indicating a high level of reproducibility in MRSI-t measurements^16^. Notably, the mean global brain temperature was 37.2 °C, although region-specific variations were identified^16^. In our subsequent investigation of patients with temporal lobe epilepsy, MRSI-t revealed significantly elevated brain temperatures in regions associated with seizure generation, as identified by electroencephalography-indicated ictal onset zones^15^. Furthermore, focal brain temperature elevations detected by MRSI-t corresponded with elevated free water detected by NODDI, suggesting that combining these techniques may provide a more comprehensive understanding of the post-acute effects of COVID-19 on the brain at both macroscopic and microscopic levels. When combined with measures of GMV, MRSI-t and NODDI may help map whether neuroinflammatory changes are associated with symptoms of PASC (e.g., cognitive and olfactory dysfunction).

Neuroimaging studies conducted on hospitalized COVID-19 patients with acute neurologic symptoms have contributed to our understanding of the neuroimaging landscape in COVID-19. However, there is a need for more neuroimaging studies that specifically investigate the neural substrates of PASC following mild COVID-19 infection, especially as symptoms can progress markedly from the symptomatic to post-acute phases. This preliminary study aims to address this research gap by analyzing MRSI-t data, along with structural and diffusion imaging data, collected from a small sample of ten otherwise healthy participants before and 3 or more months after mild COVID-19 infection. Additionally, imaging data were analyzed in conjunction with changes in mood, anxiety, and depression scores, and interpreted in conjunction with residual symptoms consistent with PASC. We hypothesized a priori that abnormalities in brain temperature, volume, and microstructure would be most pronounced in limbic and frontal regions, especially regions within or connected to the olfactory cortex (prefrontal, orbitofrontal, and insular cortices). It is important to recognize that the small sample size and preliminary nature of this study limit the generalizability of our findings. Nevertheless, by examining pre- and post-COVID-19 scans, this serendipitous dataset provides unprecedented insight into the changes that may occur after infection. By focusing on chronic olfactory dysfunction and post-acute neurological effects, this preliminary study aims to provide valuable insights into the neural substrates of PASC, particularly those that may have a greater impact on day-to-day brain function (e.g., olfaction).

## Methods

### Recruitment

Participants were initially recruited between September 2019 and February 2020 as healthy controls for the parent study, for which recruitment methods and inclusion/exclusion criteria have been previously described in detail^15,16^. The parent study aimed to assess the repeatability and reproducibility of MRSI-t-based brain temperature measurements. For that study, healthy participants aged 23 to 46 years underwent scanning at two time points, 12 weeks apart. To be included in the parent study, participants had to be able to undergo scanning at 3-Tesla and be without neurological or neuroinflammatory conditions. Women of child-bearing age were included pending a negative pregnancy test before scanning. After obtaining additional approvals from the University of Alabama at Birmingham (UAB) Institutional Review Board (IRB), participants from the parent study were recontacted to determine their eligibility for a third post-COVID-19 scan.

For the post-COVID-19 arm, all participants were scanned between May 2021 and March 2023.

Participants were included if they tested positive for SARS-CoV-2 at least three months prior (but no more than six months prior) and had mild COVID-19 symptoms that did not require hospitalization. Both participants with and without PASC symptoms were included, but all participants reported symptoms of “brain fog” either during the symptomatic or post-acute phases. Symptoms such as "brain fog," anosmia, and hypogeusia during the symptomatic or post-acute phases were considered for inclusion, with "brain fog" encompassing dysfunction in learning, memory, attention, sleep, fatigue, or mood aberrations such as increased depressive or anxiety symptoms.

Participants provided written informed consent prior to participation. All study procedures were approved by the UAB IRB and were carried out in accordance with the Declaration of Helsinki.

### Data collection and processing

#### Study visits

Participants completed three study visits. For the parent study, participants completed two study visits (T_1_ and T_2_) scheduled 12-weeks apart. Data collected at the first two visits comprised the pre-COVID-19 dataset, while the data collected at the third visit (T_3_) comprised the post-COVID-19 dataset.

#### Self-report scales and participant narratives

Data collection procedures were consistent across all three timepoints, and the methods have been previously described in detail^16^. Anxiety and depressive symptoms were measured using the Hospital Anxiety and Depression Scale (HADS), and distress was measured using the Profile of Mood States (POMS) Total Mood Disturbance (TMD) score^26,27^. Participant narratives provided information about COVID-19 infection and symptoms during both the infectious and post-infectious stages. Heart rate, blood pressure, and core body temperature were measured before scanning. Core body temperature was based on tympanic temperature measurements obtained using a Braun Pro 4000 ThermoScan aural thermometer. This was done because studies have shown that tympanic temperature can serve as a reliable indicator of brain temperature^17^. Additionally, it was important to rule out any individual core body temperature elevations that could potentially affect the accuracy of brain temperature measurements^17^.

#### Neuroimaging data acquisition

Structural, spectroscopic, and multi-shell diffusion data were collected on a 3 T Siemens Magnetom Prisma scanner using a 20-channel head coil, and processed as described in our previous studies^15,16^. T1-weighted high-resolution structural MRI data were acquired using a magnetization-prepared rapid gradient echo sequence with the following parameters: repetition time (TR) = 2400 ms, echo time (TE) = 2.22 ms, flip angle = 8°, 208 slices (0.8 mm thick), field of view = 256 mm × 256 mm. Before acquiring volumetric MRSI-t, off-resonance frequency adjustment and both automatic and manual shimming were performed to reduce frequency deviation effects and magnetic field inhomogeneities. After the 3D automatic shim, an interactive shim was performed to achieve a peak full-width half maximum (FWHM) ≤ 25 threshold. The maximum threshold for accepting the shim was FWHM ≤ 30. MRSI-t data were acquired using a 3D echo planar spectroscopic imaging sequence with the following parameters: TR1 = 1500 ms, TR2 = 511 ms, TE = 17.6 ms, lipid inversion-recovery time = 198 ms, field of view = 280 mm × 280 mm × 180 mm, voxel size = 5.6 mm × 5.6 mm × 14.4 mm (voxel volume = 451.6 mm^3^). Multi-shell diffusion MRI data were collected in 93 directions using b-values centered at 1500 and 3000 s/mm^2^ using the following parameters: TR = 3230 ms, TE = 89.2 ms, field of view = 210 × 210 mm, flip angle = 78°, 1.5 mm isotropic voxels.

#### Neuroimaging data processing

Structural images were processed by voxel-based morphometry using the Computational Anatomy Toolbox (CAT12) in Statistical Parametric Mapping (SPM12; http://www.fil.ion.ucl.ac.uk)) running in MATLAB 2021a (The MathWorks, Inc., Natick, MA, USA) as previously described^28,29^. This included skull-stripping, bias correction, tissue segmentation with partial volume estimation, denoising, normalization, modulation, and smoothing. The final modulated, smoothed volumes were statistically analyzed to investigate GMV changes over time. Total intracranial volume (TIV) was calculated using the CAT12 tissue segmentation module.

For MRSI-t data, image reconstruction and spectral processing were completed within the Metabolite Imaging and Data Analysis System (MIDAS) software package as previously described^30,31^. Data were corrected for magnetic field shifts and frequency misalignments, and signals from fat were suppressed using lipid k-space extrapolation^30^. Spectral data were reconstructed and analyzed based on prior knowledge of the resonance peaks of major metabolites^30^. Following spectral fitting, spectra were normalized and integrated with water reference data. Brain temperature in each voxel was calculated using the equation: T_CRE_ = -102.61 (Δ_H2O-CRE_) + 206.1 °C, where Δ_H2O-CRE_ = chemical shift difference between the temperature-dependent peak of H_2_O and the temperature-independent peak of the reference metabolite creatine (CRE)^30,31^. The T_CRE_ range was narrowed during processing to improve image contrast and enhance detection of subtle differences, with partial volume adjustment for grey/white matter signal intensities. T_CRE_ maps were created by interpolating the data to a 64 × 64 × 64 grid (voxel size = 4.375 mm × 4.375 mm × 5.625 mm; voxel volume = 107.7 mm^3^) spatially aligned with structural T1-weighted images.

Diffusion MRI data were corrected for eddy currents, in-scanner head motion, and EPI distortions using TORTOISE DIFF_PREP (v2.5.2b; nih.gov) and then analyzed with the NODDI Toolbox v1.01 running in MATLAB R2021a on a high-performance computing cluster^32^. NODDI analyses generated the following 3D maps: (1) orientation dispersion index (ODI), (2) fractional intracellular volume fraction (FICVF), and (3) fractional isometric volume fraction (FISO), with the contribution of the CSF compartment excluded from ODI and FICVF maps^32–39^.

#### Post-processing MRSI-t and NODDI data

T_CRE_ maps were exported from MIDAS for post-processing. To increase contrast and improve the detection of subtle differences, the T_CRE_ range was narrowed during processing, with a temperature of 32 °C scaled to 0.0 and a maximum value of 10 reflecting 42 °C^16^. When visualizing T_CRE_ data from individual participants, T_CRE_ maps were adjusted by voxelwise addition of 32 °C, resulting in spatial maps that reflected physiologically realistic values of biological relevance^16^. The resulting T_CRE_ maps underwent linear affine alignment with anatomical data using AFNI @*Align_Centers*, and were then median filtered with AFNI’s *3dMedianFilter* to reduce noise at the outer edges of the brain. They were subsequently smoothed with an 8 mm Gaussian kernel. Finally, T_CRE_ and NODDI maps were warped into a common space using the Montreal Neurological Institute (MNI) template.

### Primary olfactory cortex and secondary olfactory areas

A probabilistic atlas was used to generate parcellation masks of the primary olfactory cortex and associated secondary olfactory areas^40^. The Supplementary Figure visualizes the regions contained within these cortices. The primary olfactory cortex included the amygdala, anterior olfactory nucleus (AON), entorhinal, frontal piriform, olfactory bulb, olfactory tract, olfactory tubercle, and temporal piriform cortices. Secondary olfactory areas included: Crus II of the cerebellum, hippocampus, insula, parahippocampal cortex, thalamus, and the frontal inferior, middle, and superior orbital gyri. These masks were brought into MNI space and binarized.

For each participant’s voxelwise spatial maps (T_CRE_, FISO, FICVF, ODI, GMV), means were extracted from regions contained within the primary olfactory cortex and secondary olfactory areas. For each region, the mean voxel intensity value (T_CRE_, FISO, FICVF, ODI, GMV) was calculated by summing the intensity value for each voxel and dividing by the total number of voxels in that region. For neuroimaging data that showed pre- to post-COVID-19 changes across all participants, the percent change within each region was calculated as:

$$\% \text{Change from Pre- to Post-COVID-19 }= \frac{\text{ (Post-COVID-19 mean) - (Pre-COVID-19 mean)}}{\left|\text{Pre-COVID-19 mean}\right|}{) \times 100}$$

### Statistics

Descriptive statistics for all continuous variables were computed using GraphPad Prism version 8.0 (v.80) for Mac (GraphPad Software, La Jolla, CA, USA, https://www.graphpad.com). Repeated measures t-tests compared pre- to post-COVID-19 changes in demographic, cardiorespiratory, and mood measures.

Voxelwise repeated measures ANOVAs tested pre- to post-COVID-19 changes in T_CRE_, NODDI, and GMV maps. All repeated measures ANOVAs were performed using the Multivariate and Repeated Measures (MRM; https://github.com/martynmcfarquhar/MRM) toolbox running in MATLAB 2021a on a high-performance computing cluster. To mitigate the risk of false positives, we chose a permutation-based analysis approach for significance testing instead of employing corrections for multiple comparisons. Corrections for multiple comparisons can be overly stringent and potentially overlook meaningful effects, especially in exploratory studies with limited sample sizes. Permutation-based analyses involve systematically shuffling or rearranging the data to assess the likelihood of obtaining the observed results by chance alone. Significance was determined based on 5000 permutations, and a cluster-forming *p*-value < 0.001 was considered the threshold for statistical significance^41^. Orthogonal contrasts tested differences between post-COVID-19 data (T_3_) and pre-COVID-19 data acquired at T_1_ and T_2_.

Since brain temperature was the primary measure of interest, T_CRE_ maps were assessed alongside participants’ reports of symptoms. Difference and z-score images were created using algorithms contained within the FSL (FMRIB Software Library, version 6.0.7, https://fsl.fmrib.ox.ac.uk/) software package developed by the Oxford Centre for Functional Magnetic Resonance Imaging of the Brain (FMRIB) at the University of Oxford.

In order to visualize pre-COVID-19 T_CRE_ fluctuations, a difference image was generated using *fslmaths*:

$$\text{Pre-COVID-19} \text{T}_\text{CRE}\, \text{Fluctuations} = (\text{Pre-COVID-19} \text{T}_\text{CRE}\, \text{at} \, \text{T}_2) - (\text{Pre-COVID-19} \text{T}_\text{CRE}\, \text{at}\, \text{T}_1)$$

Post-COVID-19 T_CRE_ map increases were visualized by computing a z-score image using *fslmaths:*

$$\text{Post - COVID-19 }\text{z-map }=\left(\frac{ (\text{Post-COVID-19} \text{T}_\text{CRE}\, \text{map}) - (\text{Pre-COVID-19 mean})}{\text{(Pre-COVID-19 SD)}}\right)$$

In the equation above, pre-COVID-19 mean = mean of T_CRE_ maps collected at T_1_ and T_2_. Voxels with z ≥ 2.5 standard deviations (SDs) from the pre-COVID-19 mean were considered abnormal.

For imaging modalities that showed statistically significant differences from pre- to post-COVID, mixed-effects analyses investigated temporal and spatial variations within the a priori identified regions, including the primary olfactory cortex and associated secondary regions. Mixed-effects analyses using the restricted maximum likelihood method were performed using GraphPad Prism, with post-hoc follow-up tests to assess pairwise differences. The dependent variables for each model were mean T_CRE_, FISO, or GMV; for each modality, separate models were computed for the primary and secondary olfactory cortices. The fixed effects (type III) were brain region, time, and the interaction between brain region and time. Participant was included as a random effect. The model specification was as follows: mean [T_CRE_, FISO, or GMV] ~ region + time + region × time + (1|Participant), *p* < 0.05.

Lastly, to further investigate the presence of greater abnormalities in the primary and secondary olfactory cortices in patients with chronic olfactory dysfunction (COD), additional post-hoc tests were conducted. These tests aimed to explore region-specific differences between participants with (n = 5) and without COD (n = 5) over time. Post-hoc linear mixed-effects analyses were performed using GraphPad Prism to examine the effects of time (pre- and post-COVID-19), COD (with and without chronic olfactory dysfunction), and brain region on T_CRE_ or FISO abnormalities averaged within ROIs in the primary and secondary olfactory cortices. Participant ID was included as a random effect to account for within-subject correlation. For each dependent variable (T_CRE_ or FISO), two models were run: T_CRE_ in the (1) primary and (2) secondary olfactory cortices, as well as FISO in the (1) primary and (2) secondary olfactory cortices. Given the small number of participants with and without COD and increased risk of false positives, *p*-values were corrected for multiple comparisons using the False Discovery Rate (FDR = 0.05) with the two-stage step-up method of Benjamini and Yekutieli^42^.

### Ethical statement

The authors confirm that they have read the Journal’s position on ethical publication, and that all study procedures and this report are consistent with those guidelines.

## Results

### Pre- to post-COVID-19 changes in cardiorespiratory, mood, and global imaging measures

Of the 18 healthy adults repeatedly scanned in the parent study (pre-COVID-19), 10 adult participants (6 females, 4 males) were again scanned at T_3_ (post-COVID-19), a mean of 109 ± 24.4 days following mild COVID-19 infection^16^. Participant features, scores on mood assessments, and global imaging metrics are summarized in Table 1. Based on the time frame of infection, half of the participants were likely infected by the original SARS-CoV-2 strain (i.e., the alpha, beta, and gamma strains). The other half of participants were likely infected by later delta and omicron strains. Seven participants reported lingering “brain fog” consistent with symptoms of PASC, including increased anxiety, depression, and fatigue and decreased attention span. Five participants reported residual olfactory dysfunction (anosmia, hyposmia, or parosmia) at the time of scanning.

**Table 1:** Summary of demographic, cardiorespiratory, mood, and global imaging study measures for 10 participants scanned before and after COVID-19 infection.

			Pre-COVID-19	Post-COVID-19	Group differences
Age (years)	Mean age at study visit		27.4 ± 6.1	29.9 ± 6.6	
Cardiorespiratory metrics					
	Core body temperature (°C)		36.63 ± 0.18	36.67 ± 0.31	*t*(9) = 0.25, *p* = 0.40
	Heart rate (bpm)		79.5 ± 13.3	79.7 ± 11.4	*t*(9) = 0.05, *p* = 0.48
	Blood pressure, systolic		127.5 ± 13.6	121.2 ± 15.5	*t*(9) = 1.48, *p* = 0.09
	Blood pressure, diastolic		74.7 ± 8.2	74.9 ± 6.8	*t*(9) = 0.16, *p* = 0.44
Mood metrics					
Hospital Anxiety & Depression Scale (HADS)	HADS, depression subscale		2.4 ± 2.4	2.7 ± 2.2	*t*(9) = 0.47, *p* = 0.32
	HADS, anxiety subscale		6.0 ± 2.1	7.3 ± 3.2	*t*(9) = 1.5, *p* = 0.08
Profile of Mood States (POMS)	Tension-anxiety		7.0 ± 5.1	11.9 ± 4.3	*t*(9) = 3.32, ***p* = 0.004**
	Anger-hostility		5.6 ± 4.6	5.9 ± 3.6	*t*(9) = 0.26, *p* = 0.40
	Depression-dejection		4.9 ± 3.3	5.1 ± 3.5	*t*(9) = 0.16, *p* = 0.44
	Vigor-activity		19.2 ± 5.9	13.9 ± 6.2	*t*(9) = 3.12, ***p* = 0.006**
	Confusion-bewilderment		4.9 ± 2.7	7.0 ± 3.4	*t*(9) = 1.62, *p* = 0.07
	Fatigue-inertia		7.6 ± 4.6	10.1 ± 4.1	*t*(9) = 1.57, *p* = 0.08
	Total mood disturbance (TMD)		10.8 ± 20.6	26.1 ± 9.7	*t*(9) = 2.29, ***p* = 0.02**
Neuroimaging metrics					
Global (whole brain)	TIV (cm^3^)		1490.1 ± 173.5	1477.9 ± 169.0	*t*(9) = 0.29, *p* = 0.39
	T_CRE_ (°C)		35.70 ± 0.31	35.91 ± 0.31	*t*(9) = 1.55, *p* = 0.08
	FISO		0.12 ± 0.12	0.15 ± 0.03	*t*(9) = 2.13, ***p* = 0.03**
	FICVF		0.39 ± 0.02	0.39 ± 0.02	*t*(9) = 0.95, *p* = 0.18
	ODI		0.39 ± 0.05	0.39 ± 0.02	*t*(9) = 0.14, *p* = 0.05

Pre-COVID-19 data were collected twice, 12-weeks apart. Post-COVID-19 data were collected after resolution of infective period.

Data are represented as mean ± standard deviation. For each study measure, participants’ pre-COVID-19 data represent the mean of data collected at two timepoints before COVID-19 infection. Core body temperature was measured using a tympanic thermometer. The mean and SD for global imaging metrics were calculated across all brain regions for each set of three-dimensional spatial maps. Total intracranial volume (TIV) Brain temperature was derived from MRSI-t data, and brain morphometric measures were calculated from structural MRI images.

*bpm *beats per minute, *HADS* Hospital Anxiety and Depression Scale, *POMS* Profile of Mood States, *TMD* Total Mood Disturbance composite score on the POMS, *TIV* total intracranial volume, *MRSI-t* volumetric magnetic resonance spectroscopic imaging and thermometry, *CRE* creatine, *T*_*CRE*_ brain temperature measured by MRSI-t with CRE as the reference metabolite, *FISO* fractional isotropic volume fraction, *ODI* orientation dispersion index, *FICVF* fractional intracellular volume fraction, *GMV* grey matter volume, *olf* olfactory, *AON* anterior olfactory nucleus, *L* left, *R* right*.*

Significant values are in bold.

There were no statistically significant changes in core body temperature, heart rate, or systolic and diastolic blood pressure from pre- to post-COVID-19 (Table 1). Mood disturbance, as measured by POMS TMD scores, significantly increased from pre- to post-COVID-19 (*p* = 0.02). This increase in the POMS TMD composite score largely stemmed from the significant increase in POMS Tension-Anxiety scores (*p* = 0.004) and the significant decrease in POMS Vigor-Activity scores (*p* = 0.006).

### Brain temperature elevations, edema, and atrophy after mild COVID

**Figure 1:**
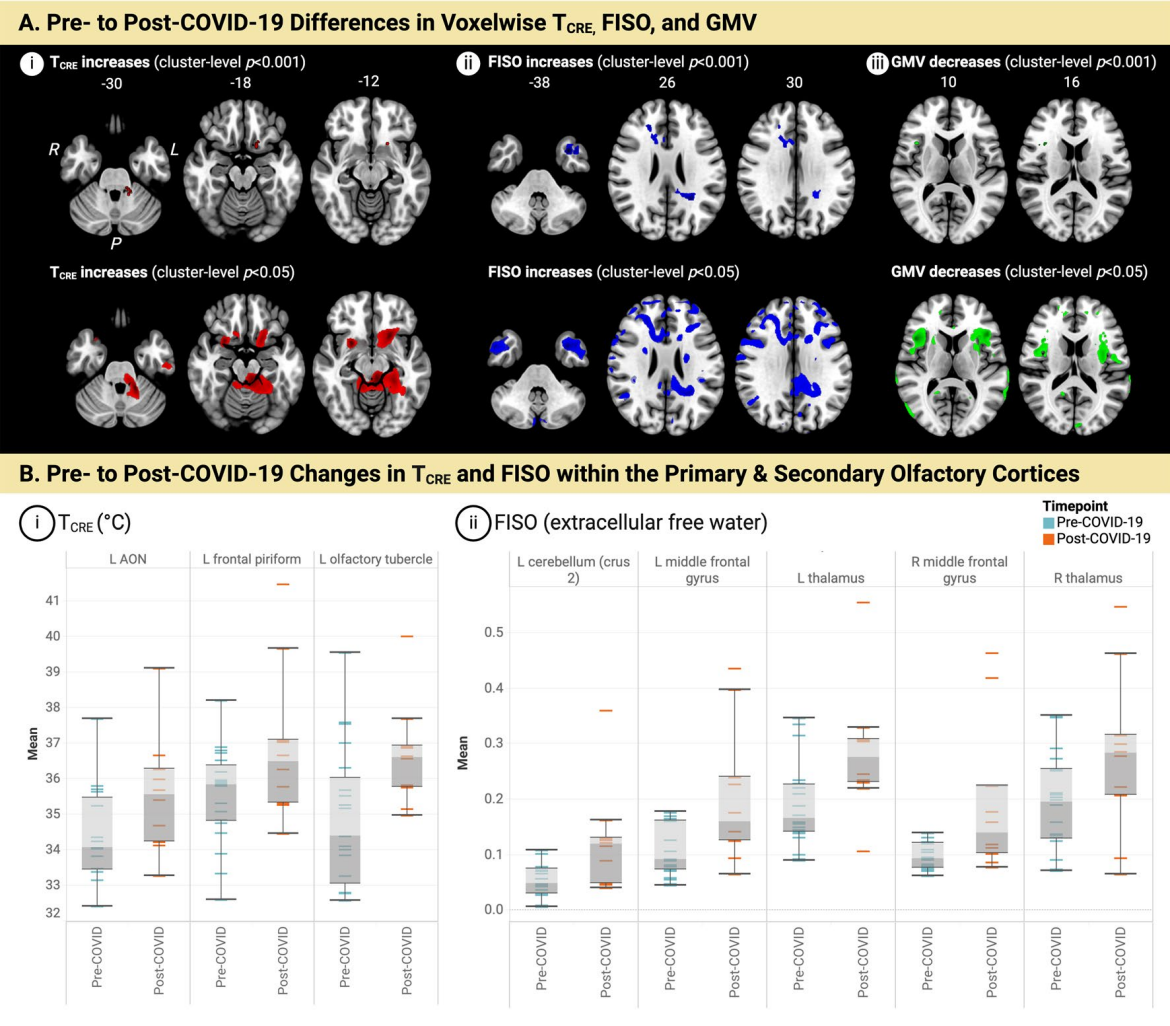
Neuroimaging metrics before and after mild COVID-19. **(A)** Voxelwise maps visualize regions showing significant pre- to post-COVID changes as determined by repeated measures ANOVAs. Brain regions showing the most pronounced post-COVID-19 effects are visualized using cluster-level *p* < 0.001 (top), with the same axial slices visualized with *p* < 0.05 (bottom). There were no changes in voxelwise FICVF or ODI. (i) Brain temperature (T_CRE_) increases (*p* < 0.001) were most pronounced in the left (L) medial orbitofrontal cortex and L inferior frontal gyrus (L IFG) pars orbitalis (top). When visualized using *p* < 0.05 (bottom), T_CRE_ increases extended to include the L inferior parietal lobule, L and right (R) superior temporal gyri, R middle cingulate cortex (MCC), and areas comprising the boundary between the L precentral and postcentral gyri. (ii) Fractional isotropic volume fraction (FISO), which measures extracellular free water (i.e., a measure of edema), significantly (*p* < 0.001; top) increased after COVID-19 in the L medial temporal pole, right (R) middle frontal gyrus, R IFG (pars Opercularis), R anterior cingulate cortex (ACC), L and R cerebellum (crus II), and R superior occipital gyrus. When visualized using *p* < 0.05 (bottom), FISO increases extended to include the L superior medial and middle temporal gyri, L paracentral lobule, as well as the L precentral and postcentral gyri; on the right, FISO increases were also found in the R precentral and angular gyri and the R inferior parietal lobule. (iii) Significant post-COVID-19 grey matter volume (GMV) decreases (*p* < 0.001) were observed in the R IFG and L cerebellar crus II (**A.iii,** top). When visualized using *p* < 0.05 (**A.iii**, bottom), GMV decreases extended more evenly across both hemispheres, in regions including the R MCC, L ACC, L and R middle temporal cortices, L and R superior frontal gyri, and L and R cerebellum (VIII). (**B**) Box-and-whisker plots illustrate the distribution and variation of T_CRE_ (left) and FISO (right) in regions of interest (ROIs) where mixed-effects analysis found pre- to post-COVID-19 increases. Boxes represent the interquartile range (IQR), which contains the middle 50% of the data. The line inside the box represents the median value, and whiskers extend to the minimum and maximum values within 1.5 times the IQR. Participants’ individual data points denote the mean for each represented by the dash symbol ("–") and are colored based on the timepoint. Pre-COVID-19 data points are shown in blue, while post-COVID-19 data points are shown in orange. In the primary olfactory cortex, (i) significant post-COVID-19 T_CRE_ elevations were predominately observed in the left hemisphere, specifically in the anterior olfactory nucleus (*p* = 0.04), frontal piriform (*p* = 0.02), and olfactory tubercle (*p* = 0.007). Additionally, statistically significant post-COVID-19 FISO increases (ii) were found in secondary olfactory areas, including crus 2 of the L cerebellum (*p* = 0.02), L and R middle frontal gyri (*p* = 0.001 for both), and L and R thalami (*p* = 0.002 and *p* = 0.003, respectively). For each plot, pre-COVID-19 data are colored in blue, while post-COVID-19 data are colored orange. *MRSI-t* volumetric magnetic resonance spectroscopic imaging and thermometry, *CRE* creatine, *T*_*CRE*_ brain temperature measured by MRSI-t with CRE as the reference metabolite, *FISO* fractional isotropic volume fraction, *ODI* orientation dispersion index, *FICVF* fractional intracellular volume fraction, *GMV* grey matter volume, *olf* olfactory, *AON* anterior olfactory nucleus, *L* left, *R* right, *P* posterior, *ROI* region of interest.

Although there was little change in global neuroimaging metrics (Table 1) averaged across all brain regions, there were significant voxelwise differences when comparing pre- to post-COVID-19. Figure 1 visualizes regions showing statistically significant abnormalities in T_CRE_, FISO, and GMV when comparing post-COVID-19 (T_3_) to pre-COVID-19 scans using both *p* < 0.001 and the less stringent *p* < 0.005 for a more comprehensive account of the findings for this small, preliminary study. Post-COVID-19 T_CRE_ elevations (*p* < 0.001) were most pronounced in the left orbitofrontal cortex and left inferior frontal gyrus (Fig. 1A.i, top; Table 2). When visualized using the less stringent *p* < 0.05 (Fig. 1A.i, bottom; Table 2), these T_CRE_ increases extended to bilateral frontotemporal regions, and areas between the left precentral and postcentral gyri (not shown). Significant post-COVID-19 increases in FISO (*p* < 0.001) were found in the frontotemporal regions and bilateral cerebellum (Fig. 1A.ii, top; Table 2). When visualized using *p* < 0.05 (Fig. 1A.ii, bottom; Table 2), FISO increases extended to include more medial limbic structures and parietal areas. Lastly, post-COVID-19 GMV decreases (*p* < 0.001) were observed in frontal and cerebellar areas (Fig. 1A.iii, top; Table 2). When visualized using *p* < 0.05 (Fig. 1A.iii, bottom; Table 2), volume losses extended more evenly across both hemispheres, and included more frontotemporal structures. Mean neurite density (FICVF) and dispersion (ODI) remained stable throughout all timepoints and all regions, even when separately considering the olfactory cortices.

**Table 2:** Summary of statistically significant group differences in voxelwise MRSI-t, NODDI, and GMV maps when comparing pre-COVID-19 to post-COVID-19 datasets.

		Spatial extent	t-value	MNI coordinates		
				X	Y	Z
Brain temperature (T_CRE_), *p* < 0.001						
Post-COVID-19 > pre-COVID	L inferior frontal gyrus (IFG), pars orbitalis	14	15.087	−20	16	−12
	L medial orbitofrontal cortex	14	7.843	−18	18	−18
	L cerebellum (4, 5)	9	20.569	−18	−42	−28
Free water (FISO), *p* < 0.001						
Post-COVID-19 > pre-COVID	L inferior temporal pole	101	36.444	−34	6	−38
	R middle frontal gyrus	221	21.076	44	10	56
	R IFG, pars opercularis	221	9.821	52	18	44
	R anterior cingulate cortex	153	13.124	4	20	28
	R superior frontal gyrus	187	17.656	26	−98	30
	L cerebellum (crus 2)	424	4.649	−38	−78	−46
	R cerebellum (crus 2)	144	5.523	30	−90	−32
Grey matter volume (GMV), *p* < 0.001						
Post-COVID-19 < pre-COVID	R IFG, pars opercularis	11	49.402	40	16	10
		11	14.907	34	16	16
	L cerebellum (crus 2)	9	26.651	−54	−60	−44

Table shows all local maxima separated by more than 5 mm for clusters containing at least 50 voxels. Regions were automatically labeled using the AAL2 atlas. x, y, and z = Montreal Neurological Institute (MNI) coordinates in the left–right, anterior–posterior, and inferior–superior dimensions, respectively.

### Pre- to post-COVID-19 abnormalities in olfactory-associated brain regions

Mixed-effects analyses demonstrated significant post-COVID-19 T_CRE_ elevations in a subset of regions contained with the primary and secondary olfactory cortices, while FISO increases were primarily detected in secondary olfactory areas.

#### *Post-COVID-19 T*_*CRE*_* elevations in the primary and secondary olfactory areas*

A mixed-effects analysis of pre- to post-COVID-19 T_CRE_ changes in the primary olfactory cortex demonstrated significant main effects of region (*F* (13, 123) = 4.95, *p* < 0.001) and time (*F* (1, 116) = 10.97, *p* = 0.001). The left hemisphere showed the most significant increases in T_CRE_ (Fig. 1B.i), with a mean increase of 1.35 °C in the left anterior olfactory nucleus (*p* = 0.04, 95% CI [0.09, 2.62]), 1.48 °C in the left frontal piriform (*p* = 0.02, 95% CI [0.21, 2.75]), and 1.75 °C in the left olfactory tubercle (*p* = 0.007, 95% CI [0.48, 3.01]). Mixed-effects analysis of T_CRE_ changes within secondary olfactory areas also found significant effects for region (*F* (15, 288) = 6.52, *p* < 0.001) and time (*F* (1, 288) = 9.39, *p* = 0.002). However, no specific brain regions showed a more pronounced effect, as indicated by the lack of a significant Region × Time interaction (*F* (13, 116) = 1.32, *p* = 0.21). Instead, subtle increases were observed across the primary and secondary olfactory cortices when comparing pre-COVID-19 (mean = 36.51°) to post-COVID-19 (mean = 36.77 °C).

#### Post-COVID-19 free water increases in secondary olfactory areas

Mixed effects analyses of pre- to post-COVID-19 FISO changes did not reveal significant changes within the primary olfactory cortex. However, analyses within the secondary olfactory cortex revealed significant main effects of region (*F* (15, 144) = 29.63, *p* < 0.001) and time (*F* (1, 144) = 48.98 *p* < 0.001). From pre- to post-COVID-19, FISO showed a mean increase ranging from 0.07 to 0.1 (Fig. 1B.ii) in crus 2 of the left cerebellum (*p* = 0.02, 95% CI [ 0.01, 0.13]), the left middle frontal gyrus (*p* = 0.001, 95% CI [0.04, 0.15], the right middle frontal gyri (*p* = 0.001, 95% CI [0.04, 0.16] ), the left thalamus (*p* = 0.002, 95% CI [0.04, 0.15]), and the right thalamus (*p* = 0.003, 95% CI [0.03, 0.14]).

### Brain temperature changes across time for each participant

**Figure 2:**
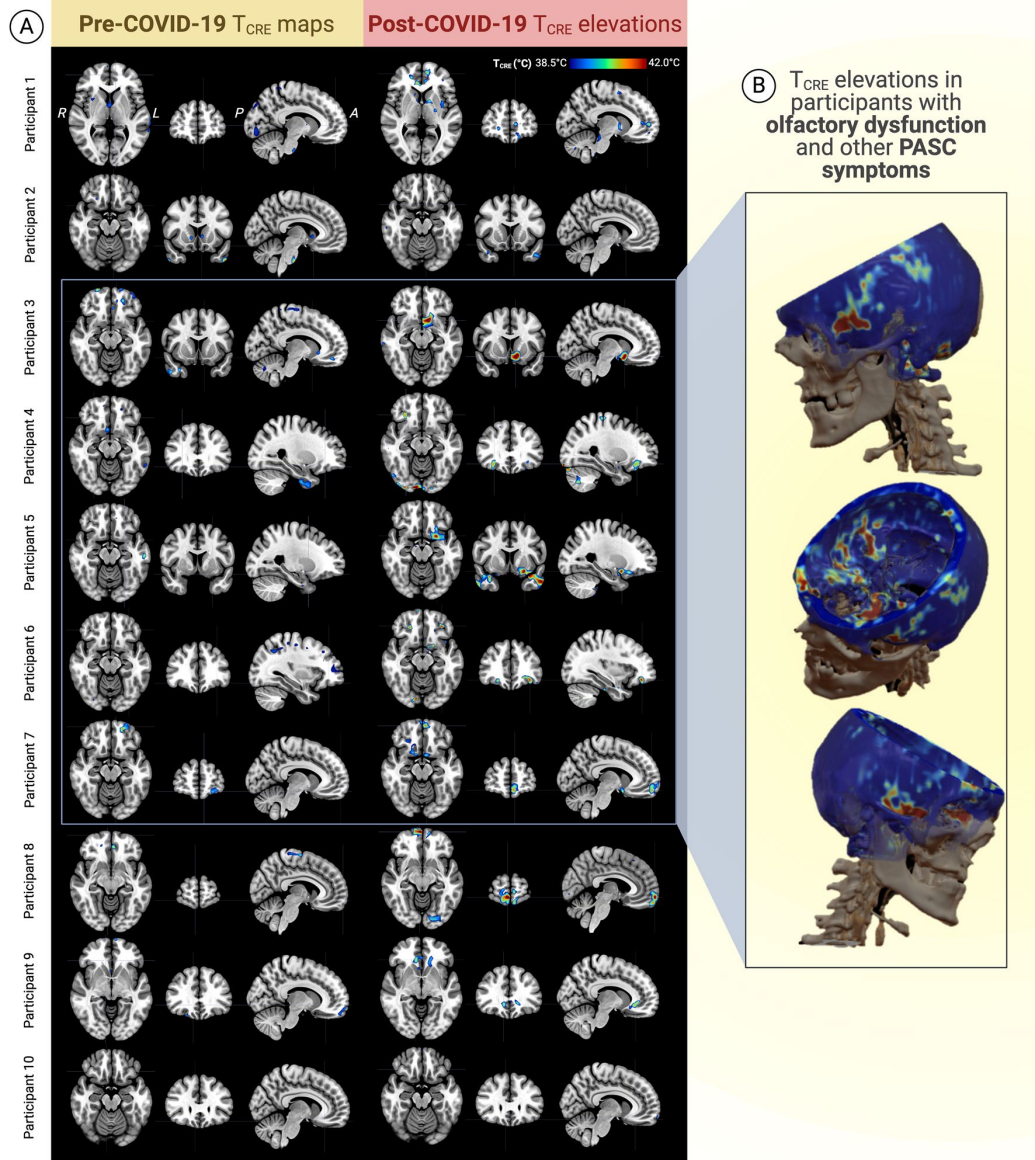
Visualization of individual participants’ brain temperature (T_CRE_) maps before and after mild COVID-19. Each participant’s pre-COVID-19 T_CRE_ maps were visualized by computing the difference between pre-COVID-19 data acquired at timepoints 1 and 2. Post-COVID-19 T_CRE_ increases were visualized using differences between a z-score image computed using each participant's pre-COVID-19 mean and standard deviation (z ≥ 2.5) and a post-COVID-19 map. (**A**) Nine out of ten participants showed post-COVID-19 brain temperature increases in olfactory, frontal, and temporal cortices. In particular, the right superior frontal (medial orbital), left inferior frontal, and right middle temporal gyri were the regions most affected across the group. Participant 10 did not show any T_CRE_ elevations or report any post-COVID-19 sequelae. (**B**) T_CRE_ increases in the olfactory cortex were observed in 5 participants (Participants 3–7, bordered in blue) who also reported post-COVID-19 olfactory dysfunction and/or other post-acute sequelae of COVID-19 (PASC). These 5 participants’ T_CRE_ maps were averaged to create the mean T_CRE_ spatial map that visualizes their T_CRE_ elevations in the primary olfactory cortex, extending to associated secondary olfactory areas. Normal T_CRE_ levels were demonstrated throughout remaining brain regions. *MRSI-t* volumetric magnetic resonance spectroscopic imaging and thermometry, *CRE* creatine, *T*_*CRE*_ brain temperature measured by MRSI-t with CRE as the reference metabolite, *L* left, *R* right, *PASC* post-acute sequelae of COVID-19.

Figure 2 visualizes typical (i.e., pre-COVID) T_CRE_ fluctuations in each participant’s pre-COVID-19 data (T_1_ and T_2_) as compared to post-COVID-19 (T_3_) T_CRE_ elevations. These difference images and z-maps were evaluated alongside participants’ narrative reports of PASC symptoms, with T_CRE_ ≥ 38.5 °C (2.5 SDs from the healthy mean) considered abnormal elevations.

1. Participant 1 reported post-COVID-19 declines in attention and working memory, but did not experience olfactory dysfunction. Frontal T_CRE_ increases were found in the right posterior orbitofrontal cortex, bilateral anterior cingulate cortex, and left superior frontal gyrus. Right temporal T_CRE_ increases were mainly in the superior temporal pole and hippocampus (mean T_CRE_, right hippocampus = 38.78 °C), the latter being a component of the secondary olfactory cortex.
2. Participant 2 reported post-COVID-19 "fogginess" and anxiety, and showed T_CRE_ elevations in the frontal and temporal cortices. These included the bilateral superior frontal gyri, left middle temporal pole and right inferior temporal gyrus.

Participants 3–7 had T_CRE_ increases within olfactory regions and reported olfactory dysfunction and other symptoms of PASC (Fig. 2A, B).

3. Participant 3 reported post-COVID-19 hyposmia, with T_CRE_ elevations in the left olfactory cortex and the right superior frontal, left inferior frontal, and right middle temporal gyri.
4. Participant 4 reported post-COVID-19 hyposmia and fatigue, and demonstrated T_CRE_ elevations in the left middle temporal gyrus and right fusiform.
5. Participant 5 experienced post-COVID-19 hyposmia, decreased attention, and increased anxiety and fatigue. T_CRE_ increases were found in the left inferior frontal gyrus and bilateral temporal lobe.
6. Participant 6 experienced post-COVID-19 parosmia and fatigue. T_CRE_ increases were detected in the left inferior and superior frontal gyri, bilateral temporal lobe, and olfactory areas.
7. Participant 7 reported post-COVID-19 parosmia and memory issues, but no anosmia during the infectious stage. T_CRE_ elevations were found in the medial, superior, and inferior frontal gyri, extending to the olfactory cortex.

Participants 8 to 10 showed fewer regions with T_CRE_ and FISO water abnormalities, and reported few—if any—symptoms of PASC.

8. Participant 8 recovered from peri-COVID-19 anosmia beyond the infection period, but experienced post-COVID-19 parosmia. T_CRE_ elevations were found in the frontal and cerebellar lobes, particularly in the right medial frontal gyrus.
9. Participant 9 did not have post-COVID-19 olfactory dysfunction or other post-COVID-19 sequelae. However, they did show T_CRE_ elevations in the left middle frontal gyrus and bilateral anterior cingulate.
10. Participant 10 showed typical T_CRE_ values. During their five-day infection period, they reported hyposmia, fatigue, and worse mood; however, they did not experience any long-term post-COVID-19 symptoms.

### Comparison of participants with and without chronic olfactory dysfunction

The post-hoc test comparing the T_CRE_ in the primary olfactory cortices for participants with COD vs. participants without COD, before and after COVID-19, revealed a significant interaction for COD × Time (*F*(1, 96) = 71.55, *p* < 0.0001). Follow-up simple effects tests showed this difference was significantly pronounced in the left olfactory tubercle, where participants with COD had a mean T_CRE_ increase of 3.34 (*p* = 0.0001, adjusted *p*_FDR_ = 0.0189, 95% CI [1.42, 5.27]). Two other effects were significant at *p* < 0.05 but did not survive FDR-correction: 1) a mean increase in T_CRE_ of 2.20 in the left amygdala (*p* = 0.04, 95% CI [0.94, 3.47]) from pre- to post-COVID, and 2) a mean T_CRE_ increase of 1.93 in the right olfactory tubercle (*p* < 0.001, 95% CI [1.38, 2.47]). No significant results were found for the analysis comparing FISO before and after COVID-19 for participants with and without COD.

**Figure 3:**
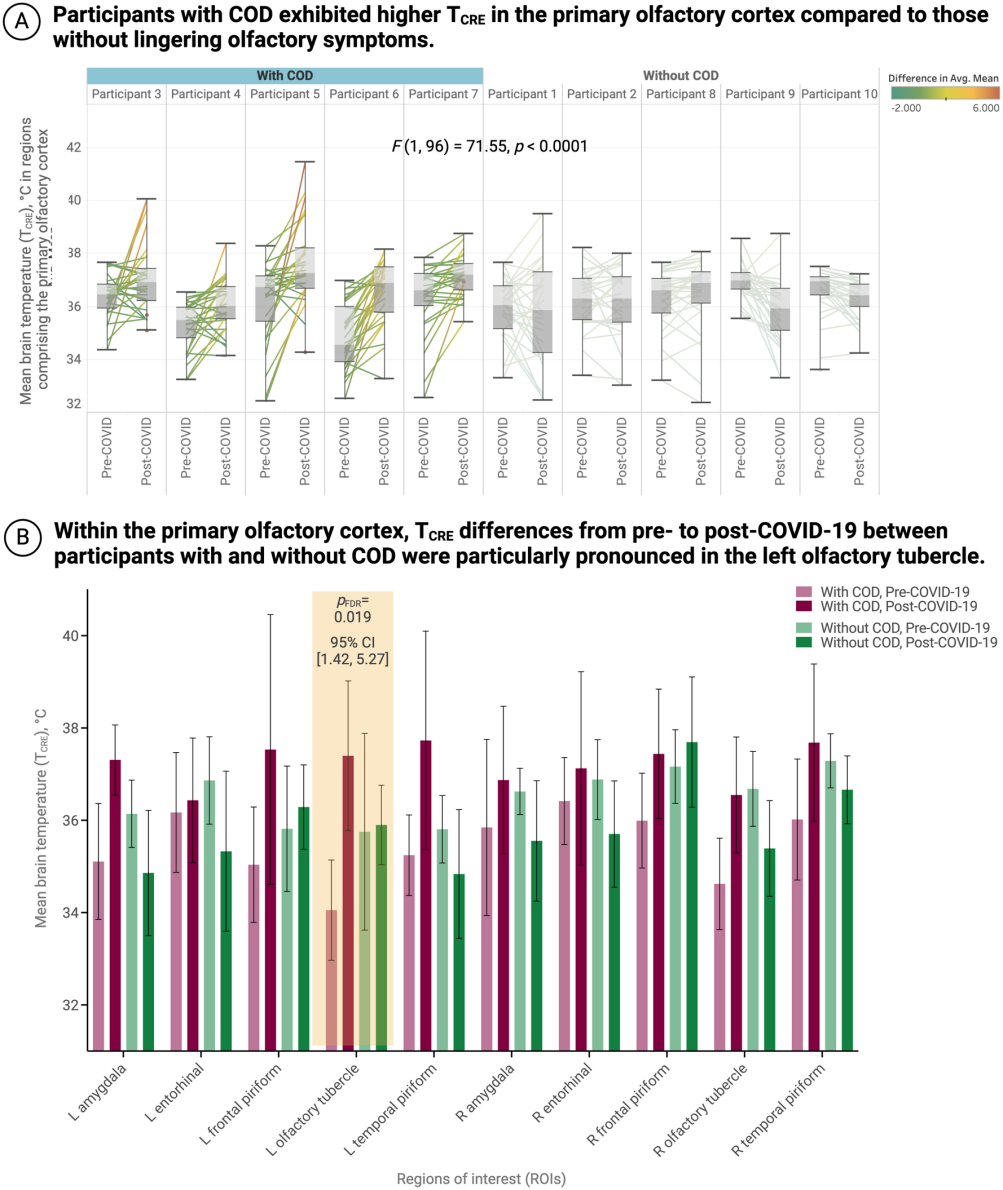
Participants with chronic olfactory dysfunction (COD) had increased T_CRE_ in the left olfactory tubercle 3 months after mild COVID-19 infection when compared to participants without lingering olfactory symptoms. **(A)** In the primary olfactory cortex, participants with COD exhibited significantly higher T_CRE_ compared to those without COD (*p* < 0.0001). The box and whisker plots illustrate the distribution and variability of T_CRE_ in the primary olfactory cortex before and after COVID-19, and are categorized by the presence or absence of chronic olfactory dysfunction. The lines connecting the pre- and post-COVID-19 boxes represent the difference in mean T_CRE_ for each region of interest (ROI) in the primary olfactory cortex, ranging from the least difference (green) to the greatest difference (orange). (**B**) Within the primary olfactory cortex, participants with COD showed a significant T_CRE_ increase (mean difference = 3.34 ℃) in the left olfactory tubercle (adjusted *p*_FDR_ = 0.0189, 95% CI [1.42, 5.27]). Bar plots visualize mean T_CRE_ in participants with and without COD in regions within the primary cortex that showed a tendency of change from pre- to post-COVID-19. Data for participants with COD are visualized in red, while data for those without COD are visualized in green; pre-COVID-19 data are lighter while post-COVID-19 data are darker. The finding in the left olfactory tubercle, which survived corrections for multiple comparisons using the False Discovery Rate (FDR), is highlighted in yellow. *COD* chronic olfactory dysfunction, *CRE* creatine, *T*_*CRE*_ brain temperature measured by MRSI-t with CRE as the reference metabolite, *L* left, *R* right, *ROI* region of interest, *CI* confidence interval, *FDR* false discovery rate.

These post-hoc findings are visualized in Fig. 3. The top part of the figure (Fig. 3A) displays the mean T_CRE_ differences across the entire primary olfactory cortex in participants with and without COD, addressing the question of whether there is a general T_CRE_ difference in the primary olfactory cortex between the two groups. The bottom part of the figure (Fig. 3B) illustrates the regions where T_CRE_ increased from pre- to post-COVID-19 when comparing participants with and without COD, providing information on the specific locations where these differences are most prominent.

## Discussion

### Main findings

This study is the first to investigate the effects of COVID-19 on brain temperature, microstructure, and brain volume in participants who were scanned before and after experiencing mild COVID-19 infection with or without PASC. By comparing datasets acquired before and after the infection, we gain valuable insights into how multimodal imaging evolved over time in a small sample of young and otherwise healthy individuals, free from the confounding factor of cognitive aging. Our findings shed light on the potential neurological consequences of mild COVID-19, with increased brain temperature and signs of atrophy observed post-infection. Notably, the primary olfactory cortex showed more pronounced T_CRE_ changes in individuals reporting loss of smell, suggesting some individuals may have a distinct susceptibility to these effects after infection. These findings underscore the need for individual-level analysis and long-term monitoring of COVID-19 patients, as even mild cases may have prolonged neurological effects or PASC.

In the following sections, we delve into the specific findings and explore the mechanisms behind them, including inflammation, vascular changes, and direct viral invasion. Additionally, we discuss the implications for the ongoing management of individuals with mild COVID-19 infections.

### Post-COVID-19 brain temperature and free water increases

Increased T_CRE_ and FISO, as well as decreased GMV, were identified post-COVID-19 infection, with the most pronounced increases in frontal regions including the olfactory cortex (*p* < 0.001). Prior studies have shown similar GMV reductions in frontal and temporal areas in older patients or in patients with a history of severe infection^43^. The present study found evidence suggestive of post-COVID-19 edema (FISO) and brain atrophy (GMV) in the right inferior frontal gyrus, while increased brain temperature was found in the left inferior frontal gyrus. This asymmetry may be due to subtle increases in T_CRE_ that did not reach the threshold for statistical significance, especially as T_CRE_ fluctuations are much more subtle due to the brain’s high heat capacity^19^. Regardless, the convergence of abnormalities in the inferior frontal gyrus indicates that pathophysiology is occurring in a region that is central to cognition. At a less-stringent threshold of *p* < 0.05, the pattern of T_CRE_, FISO, and GMV abnormalities was more diffuse and bilateral, extending to involve several frontal regions, as well as the middle temporal cortices. This may be an indication that more subtle and widespread changes may be driving PASC symptoms, beyond those detected by the more stringent *p* < 0.001 threshold.

Pre- to post-COVID-19 changes within the olfactory cortices were only observed in T_CRE_ and FISO data. T_CRE_ abnormalities in the olfactory cortex, particularly in the primary olfactory cortex, were most pronounced in participants with on-going olfactory dysfunction and other chronic post-COVID-19 sequelae. Two regions in particular, the left olfactory tubercle and the left frontal piriform cortex, showed significant increases in T_CRE_. The olfactory tubercle receives input from the olfactory bulb and plays a crucial role in transforming olfactory information into behaviorally relevant neural codes, particularly in the context of odor-guided eating behaviors^44,45^. Moreover, the frontal piriform cortex is a vital region for representing odors, allowing for odor discrimination and olfactory working memory^46–48^. Abnormal brain temperature and edema in patients with PASC may indicate chronic pathophysiology in these structures, resulting in difficulty recognizing and discriminating odors, and difficulties with olfactory memory, which can manifest as the chronic anosmia, hyposmia, or parosmia seen in PASC patients. Importantly, the left olfactory tubercle also showed the most pronounced T_CRE_ changes when comparing participants with and without COD (Fig. 3). Participants with COD also had higher T_CRE_ than those without ongoing parosmia or anosmia within the left amygdala and right olfactory tubercle, but these effects did not survive corrections for multiple comparisons. Still, it is worth mentioning that the amygdala plays a critical role in monitoring olfactory cues and, in conjunction with the hippocampus, creating odor-based episodic memories. The amygdala’s dual roles in fear and memory may be additionally impacted by COVID-induced biochemical alterations. This may explain how the virus simultaneously causes olfactory dysfunction, aberrant mood, and both anxiety and depressive symptoms, all of which occupy the spectrum of symptoms collectively referred to as “brain fog.”

Lastly, FISO increases and GMV decreases were found in the cerebellum (Fig. 1A), indicating evidence of increased free water (i.e., edema) and brain atrophy. These findings are consistent with prior literature indicating abnormalities in olfactory and limbic regions extending to the cerebellum^10,49^. Retrospective ^18^F-FDG PET studies have revealed cerebellar hypometabolism in long COVID-19 patients, associated with olfactory dysfunction (hyposmia/anosmia) and cognitive impairments^43^. Anosmia and ageusia have been linked to cerebellar lesions, due to the cerebellum’s mediation of olfactory-related responses to unpleasant odors^50,51^. The cerebellum modulates memory storage and retrieval, particularly episodic memory retrieval and the storage of emotional information such as fear memories^52–54^. Additionally, acute and chronic stressors can contribute to brain and bodily inflammation and depression, and lockdown itself can introduce stress-related brain volume reductions^55–57^. Thus, the cerebellar abnormalities detected may be reflective of the pandemic’s effects rather than a representation of a brain region mediating PASC symptoms.

### Neurobiological phenomena underlying post-COVID-19 neuroimaging abnormalities

**Figure 4:**
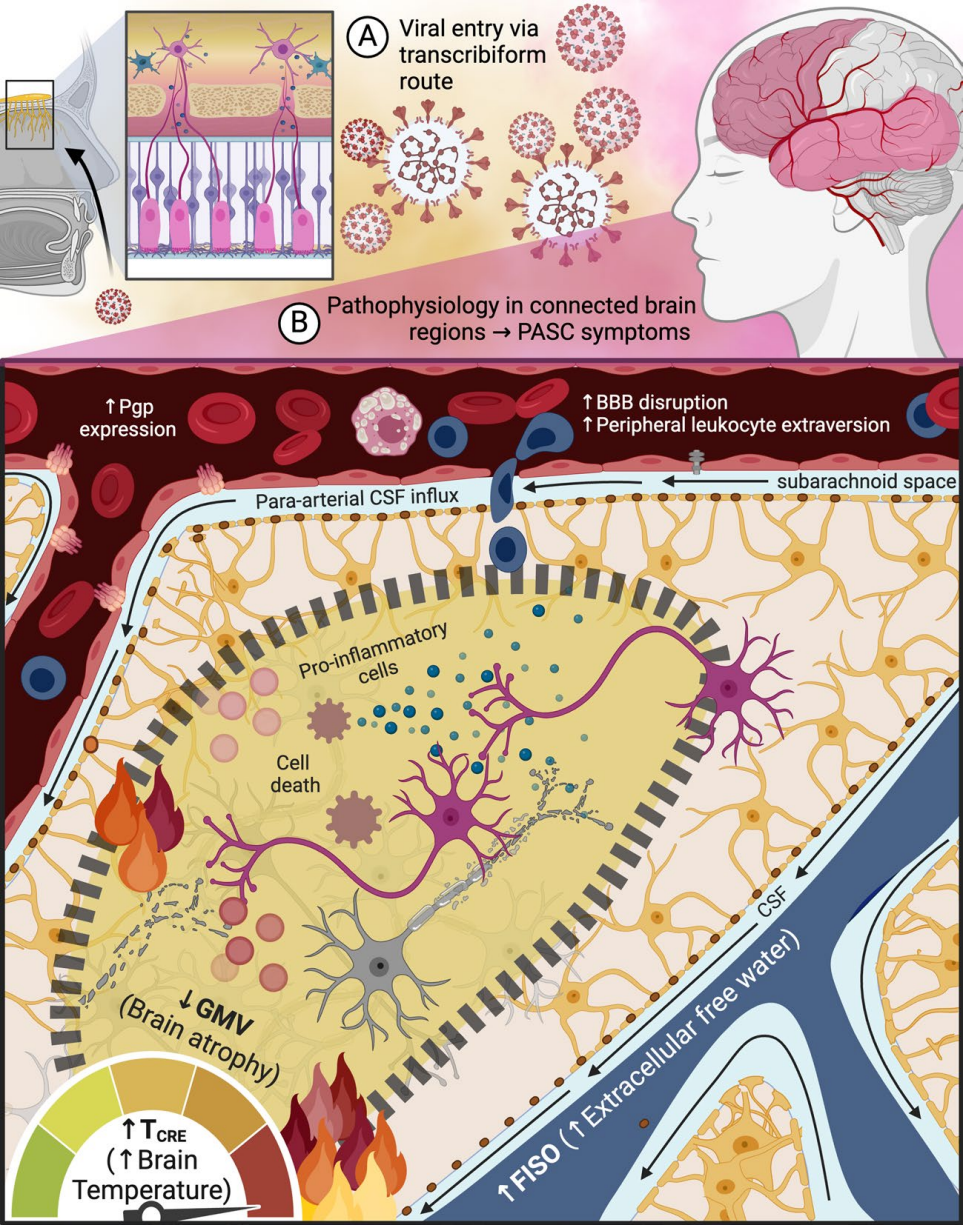
The potential entry of the SARS-Cov-2 virus into the brain through the transcribiform pathway may contribute to neuroinflammation during both the (**A**) acute and (**B**) chronic phases of mild COVID-19 infection. This hypothesis suggests that viral entry could activate sustained microglial cell activation, leading to cell death and edema. These changes may manifest as increased brain temperature (T_CRE_) and extracellular free water (fractional isotropic volume fraction or FISO), which are proposed indicators of neuroinflammation. While the imaging data in this study support this hypothesis, the study design is not suitable for establishing causal relationships. Future investigations should explore the potential connection between SARS-Cov-2 and neuroinflammation, as well as the mechanisms underlying the virus's interaction with the brain and the long-term effects of COVID-19 on neurological function. This figure was created using Biorender.com. *MRSI-t* volumetric magnetic resonance spectroscopic imaging and thermometry, *CRE* creatine, *T*_*CRE*_ brain temperature measured by MRSI-t with CRE as the reference metabolite, *FISO* fractional isotropic volume fraction, *GMV* grey matter volume, *BBB* blood brain barrier, *Pgp* P-glycoprotein, *CSF* cerebrospinal fluid.

The mechanisms underlying the observed neuroimaging findings and PASC are complex and multifaceted. In patients with respiratory symptoms, the acute phase of COVID-19 infection can lead to mucus production and swelling of the mucosa, resulting in airway narrowing and nasal cavity blockage. This mechanical obstruction may cause acute peri-COVID-19 anosmia. Additionally, the virus has the potential to travel through the transcribiform pathway, triggering inflammation and neuronal damage in the olfactory epithelium (Fig. 4A)^58^. Persistent inflammation in the chronic phase or its spread to interconnected brain regions may lead to sustained microglial activation, cell death, and edema in secondary olfactory areas (Fig. 4B) contributing to the long-term neurological symptoms associated with PASC. The persistent neuroinflammation induced by COVID-19 can result in prolonged symptoms such as cognitive dysfunction, fatigue, and depression, even after the infection has cleared^59,60^.

It is important to note that chronic neuroinflammation is not unique to COVID-19 and has been observed in conditions of chronic stress. Previous research has linked such neuroinflammation to abnormalities in the primary and secondary olfactory cortices and the amygdala^61,62^. Additionally, T_CRE_ and FISO abnormalities were identified in the piriform cortex, a region highly connected to the amygdala and insula, which plays a key role in fear-based memories and behaviors^63^.

Our study contributes to the evolving narrative surrounding PASC and post-COVID-19 neuroimaging abnormalities. It underscores the need for ongoing research to fully elucidate the various mechanisms involved. Importantly, our proposed mechanism aligns with the broader literature on the neuroinflammatory effects of COVID-19^11–14,64^. While our work adds valuable insights, we recognize that it is just one perspective on this intricate issue. It is worth noting that multiple potential mechanisms, such as neurotropism and genetic predisposition, may play a role—and the presence, nature, and severity of PASC symptoms may be more individualized and require personalized intervention^65^. Future studies should continue exploring these multifaceted aspects to comprehensively grasp the complexities that arise after mild COVID-19 infection.

### Study limitations and future studies

The study was strengthened by its short-term longitudinal design, which allowed assessing multimodal changes over time. The inclusion of young, healthy participants who experienced mild COVID-19 provided insight into virus’ impact on cognitive functioning in those less likely to suffer from severe symptoms or cognitive aging. However, while this study population fills a gap in the literature, the findings may not be generalizable due to the small sample (N = 10) and the inclusion of only young participants who contracted mild COVID. In addition, a single post-COVID-19 dataset was collected for each participant, which may not be sufficient to characterize post-COVID-19 changes in the brain over time. Lastly, the absence of a control group makes it difficult to definitively confirm that the observed changes were caused by COVID-19 infection. However, the collection of two pre-COVID-19 scans and the previously documented stability of MRSI-t and NODDI provide confidence that the post-COVID-19 findings are meaningful^16,66^.

Although we employed permutation-based analyses to mitigate the risk of false positives, the decision not to apply corrections for multiple comparisons may be considered a limitation, given the potential for increased Type I error rates in exploratory studies. The variability in scanning intervals between pre- and post-COVID-19 imaging sessions introduces a potential confounding factor, as the duration of time between scans may influence observed changes in brain temperature, structure, and microstructure. While our study addresses the neuroimaging consequences of mild COVID-19 infection, it does not delve into the potential interplay of various treatment modalities or interventions, which could be an avenue for future research. The reliance on self-reported symptoms and mood assessments introduces subjectivity into our study, and the lack of objective measures for certain variables may impact the precision of our correlations and interpretations. Lastly, the exploratory nature of our study and the inclusion of a broad range of neuroimaging parameters may increase the risk of chance findings, emphasizing the need for further validation in larger, more controlled investigations.

Participants with imaging before and after mild COVID-19 present a valuable opportunity to study the neural substrates of PASC symptoms, and the timeline of data collection is undoubtedly a major strength of the study. However, it is difficult to discern the effects of the two-year pandemic and lockdown on the brain from the effects of COVID-19, especially as it shares similar symptoms with the common cold. This study has demonstrated T_CRE_, FISO, and GMV abnormalities in mild infection, which may be a result of the extreme psychological distress caused by the pandemic or a consequence of the virus itself. To understand the neurobiological and psychological remnants of mild-COVID-19 infection, as well as the effects of the pandemic, further research is necessary. Future studies should investigate the long-term neurological consequences of mild COVID, the impact of PASC on specific groups, and if PASC is a result of COVID-19 or a byproduct of the stress and isolation of the pandemic. Although there is evidence that mild COVID-19 and pandemic-induced stress both cause neurobiological changes and associated symptoms, further research is needed to better understand these effects. It is also important to compare those who have recovered quickly from COVID-19, or were asymptomatic, to those who suffer from PASC.

## Conclusion

In conclusion, we found post-COVID-19 brain temperature elevations and edema in the primary and secondary olfactory cortices. These changes were most pronounced in participants with post-COVID-19 olfactory dysfunction and chronic “brain fog” symptoms such as increased anxiety, depression, and fatigue, and decreased attention. This supports the notion that even mild COVID-19 infections can cause long-term changes in the brain's structure and physiological function. Furthermore, our findings indicate that mild COVID-19 may lead to subtle neuroinflammatory changes and associated symptoms, even if these symptoms are not initially present during the peri-infectious stage. It remains unclear if the changes seen with PASC are specific to SARS-CoV-2. If regions within the primary and secondary olfactory cortices are particularly vulnerable to post-infectious changes, these insights may apply to other viruses that impact the olfactory cortex. Further research is needed to elucidate the mechanisms underlying these phenomena, especially whether these changes are caused by the virus, the pandemic, or both.

### Supplementary Information

Supplementary Figure 1.

Supplementary Legends.

## Data Availability

Deidentified data will be made available upon written request pending data sharing agreement.
