# Supplementary figures and images for: Brain temperature and free water increases after mild COVID-19 infection
Source: Sci Rep. 2024 Mar 28;14:7450. doi: 10.1038/s41598-024-57561-6 (PMC10978935; doi:10.1038/s41598-024-57561-6)

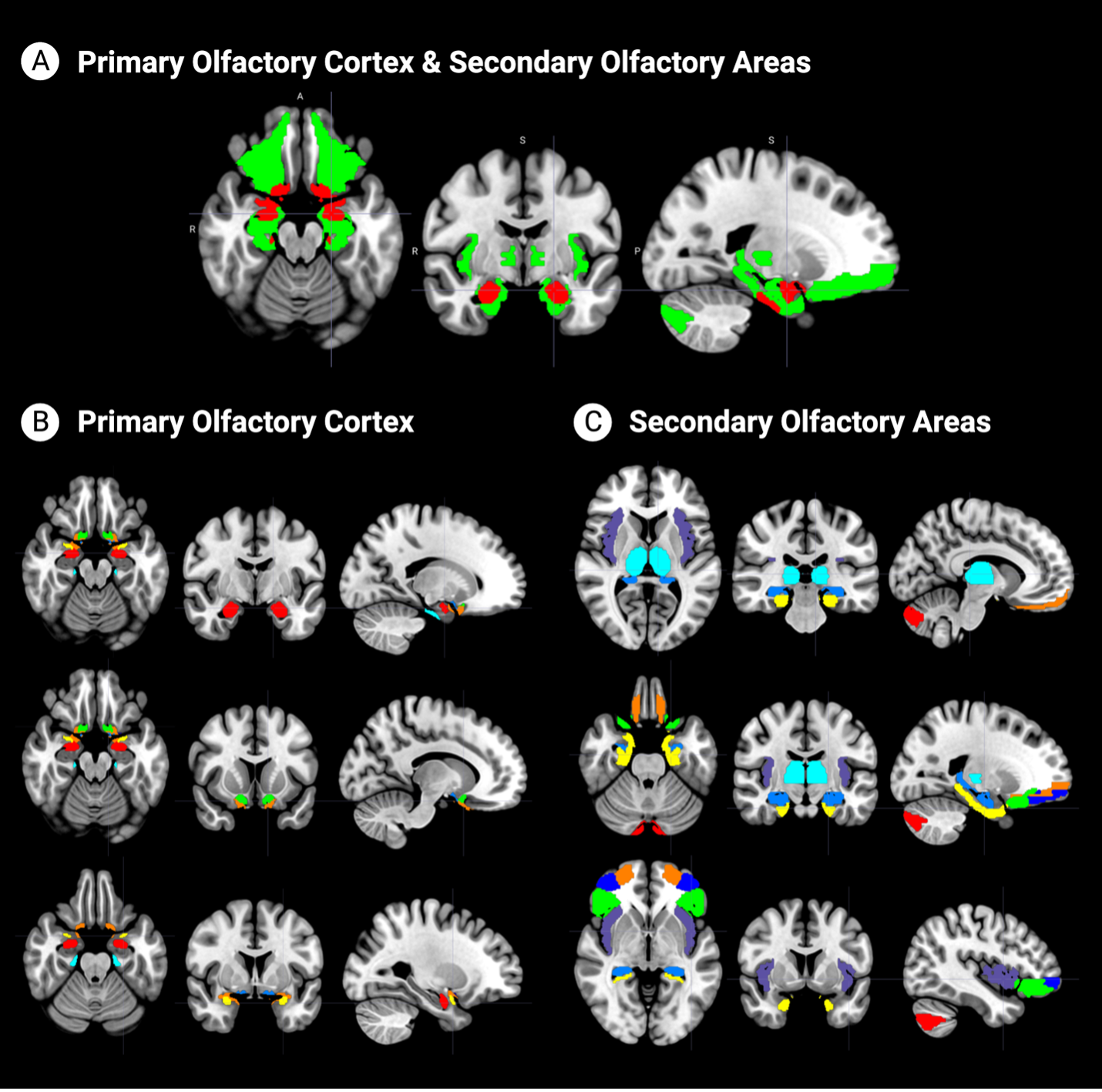

Supplement: Supplementary file 1 — Supplementary Figure 1. [file 41598_2024_57561_MOESM1_ESM.png]
