# Supplementary material for: Brain temperature and free water increases after mild COVID-19 infection
Source: Sci Rep. 2024 Mar 28;14:7450. doi: 10.1038/s41598-024-57561-6 (PMC10978935; doi:10.1038/s41598-024-57561-6)
Supplement: Supplementary file 2 — Supplementary Legends. [file 41598_2024_57561_MOESM2_ESM.docx]

**Supplementary Figure. Masks used to extract mean T_CRE_ and FISO in regions contained within the primary olfactory cortex and associated secondary regions.**

1. The primary olfactory cortex (red) and secondary olfactory areas (green) visualized together.
2. The primary olfactory cortex included the bilateral amygdala (blood red), anterior olfactory nucleus (AON; green), entorhinal cortex (light blue), frontal piriform (plasma purple), olfactory bulb (winter blue), olfactory tract (orange), olfactory tubercle (cool blue), and temporal piriform (yellow).

The secondary olfactory areas included Crus 2 of the cerebellum (red), frontal inferior (green), middle (blue), and superior (orange) orbital gyri, hippocampus (periwinkle blue), insula (purple), parahippocampal cortex (yellow), and thalamus (light blue).
